# Melatonin enhances autologous adipose-derived stem cells to improve mouse ovarian function in relation to the SIRT6/NF-κB pathway

**DOI:** 10.1186/s13287-022-03060-2

**Published:** 2022-08-04

**Authors:** Qiao-yi Huang, Shao-rong Chen, Yun-xia Zhao, Jia-ming Chen, Wei-hong Chen, Shu Lin, Qi-yang Shi

**Affiliations:** 1 Department of Gynaecology and Obstetrics, Second Affiliated Hospital of Fujian Medical University, No. 34 North Zhongshan Road, Quanzhou, 362000 Fujian Province China; 2 Department of Gynaecology and Obstetrics, University of Hong Kong-Shenzhen Hospital, Shenzhen, China; 3 Centre of Neurological and Metabolic Research, Second Affiliated Hospital of Fujian Medical University, No. 34 North Zhongshan Road, Quanzhou, 362000 Fujian Province China; 4 Group of Neuroendocrinology, Garvan Institute of Medical Research, 384 Victoria St, Sydney, Australia

**Keywords:** Autologous adipose-derived stem cells, Melatonin, Premature ovarian insufficiency, SIRT6/NF-κB pathway

## Abstract

**Background:**

Premature ovarian insufficiency (POI) is the main cause of female infertility. Adipose-derived stem cells (ADSCs) are ideal candidates for the treatment of POI. However, some deficient biological characteristics of ADSCs limit their utility. This study investigated whether melatonin (MLT)-pretreated autologous ADSCs were superior to ADSCs alone in the treatment of the POI mouse model.

**Methods:**

Autologous ADSCs were isolated and cultured in MLT-containing medium. Surface markers of ADSCs were detected by flow cytometry. To determine the effect of MLT on ADSCs, CCK-8 assay was used to detect ADSCs proliferation and enzyme-linked immunosorbent assay (ELISA) was used to detect the secretion of cytokines. The POI model was established by intraperitoneal injection of cyclophosphamide and busulfan. Then, MLT-pretreated autologous ADSCs were transplanted into mice by intraovarian injection. After 7 days of treatment, ovarian morphology, follicle counts, and sex hormones levels were evaluated by hematoxylin and eosin (H&E) staining and ELISA, and the recovery of fertility was also observed. The expressions of SIRT6 and NF-κB were detected by immunohistochemical (IHC) staining and quantitative real-time polymerase chain reaction (qRT-PCR).

**Results:**

Flow cytometry showed that autologous ADSCs expressed CD90 (99.7%) and CD29 (97.5%). MLT can not only promote the proliferation of ADSCs but also boost their secretory function, especially when ADSCs were pretreated with 5 µM MLT for 3 days, improving the interference effect. After transplantation of autologous ADSCs pretreated with 5 µM MLT, the serum hormone levels and reproductive function were significantly recovered, and the mean counts of primordial follicle increased. At the same time, the expression of SIRT6 was remarkably increased and the expression of NF-κB was significantly decreased in this group.

**Conclusions:**

MLT enhances several effects of ADSCs in restoring hormone levels, mean primordial follicle counts, and reproductive capacity in POI mice. Meanwhile, our results suggest that the SIRT6/NF-κB signal pathway may be the potential therapeutic mechanism for ADSCs to treat POI.

## Introduction

Premature ovarian insufficiency (POI) is a reproductive endocrine disease characterized by reduced ovarian follicles and hormone secretion disorders and manifested by menstrual disorders, infertility, and menopausal syndrome before 40 years of age [1, 2]. In addition, POI is a predisposing factor for many health complications, such as increased lifetime risk for sexual dysfunction, osteoporosis, and cardiovascular and neurocognitive impairments. Although causative factors include genetic factors, autoimmunity, viral infection, iatrogenic factors, and environmental factors, POI is mainly idiopathic [3]. The clinical treatment option for patients with POI is hormone replacement therapy (HRT). However, HRT cannot fundamentally restore ovarian function and is accompanied by various side effects. Therefore, some other potential therapeutic approaches are being explored.

Progress in using mesenchymal stem cells (MSCs) for the treatment of POI has been reported [4]. MSCs are a promising therapeutic approach for POI. Notably, although MSCs have less immunogenicity, allogeneic MSCs can still elicit immune responses leading to rejection [5]. By comparison, autologous MSCs have a lower risk of initiating an immune response and a lower incidence of morbidity [6, 7]. Among the various MSCs, adipose-derived stem cells (ADSCs) have been widely used because of their many advantages. ADSCs can be largely extracted from subcutaneous adipose tissue, with low immunogenicity, less ethical controversy, and stably proliferative capacity [8, 9]. Studies have shown that transplantation of autologous ADSCs can restore ovarian hormone levels and improve ovarian function [10]. Currently, underlying mechanisms of stem cell-mediated restoration of ovarian functions have not been fully elucidated. The main therapeutic mechanisms include migration, homing, immune regulation, and paracrine stimulation. Several studies have revealed that stem cells might improve ovarian function through the paracrine mechanism [11, 12]. However, detailed mechanisms need further investigation. Therefore, the focus of determining the therapeutic effect is on exploring the potential and mechanism of MSCs.

Melatonin (*N*-acetyl-5-methoxytryptamine, MLT) is a neurohormone mainly produced by the pineal gland that is also synthesized in reproduction-related tissues such as ovaries and testes [13, 14]. MLT is a key mediator for circadian rhythms and has powerful immunomodulatory, antioxidant, and anti-aging functions [15]. The potential protective effect of MLT on ovarian tissue has also been investigated; it has been shown that MLT protects primordial follicles from cyclophosphamide (CTX)-induced damage and delays ovarian aging [16, 17]. Moreover, MLT may be conducive to follicle development in the state of inflammation [18]. In addition, MLT plays an important role in the proliferation, differentiation, and survival of MSCs [19, 20]. In this study, ADSCs were cultured in vitro in MLT-containing medium, and the results showed that MLT promoted the proliferation of ADSCs.

Sirtuin 6 (SIRT6), a member of the sirtuins family, is a key regulator of cellular metabolism, genomic stability, DNA repair, and aging [21]. Previous studies have indicated that SIRT6 inhibited the overactivation of primordial follicles and prolonged the ovarian function lifespan, while SIRT6-deficient-mice developed premature aging syndromes [22, 23]. In addition, transcription factor nuclear factor kappa B (NF-κB) is a key regulator of apoptosis, inflammation, immunity, and aging. It is involved in the occurrence and development of POI to a certain extent, which has been confirmed in POI animal models [24, 25]. Accumulated evidence indicates that SIRT6 inhibits certain NF-κB target genes by phosphorylating TAK1 and deacetylating H3K9 in the promoter regions [26]. The SIRT6/NF-κB signaling pathway plays a positive regulatory role in a variety of inflammation, tumor, and aging-related diseases. Currently, the role of this pathway in POI has not been investigated.

Both ADSCs and MLT have ovarian repair functions, and we hypothesized that combined use of the two could effectively alleviate ovarian damage. In this study, we aimed to investigate the beneficial effects and potential mechanisms of the MLT-pretreated autologous ADSCs in the POI mouse model.

## Materials and methods

### Experimental animals

Six-week-old female C57/BL6 mice were purchased from the Experimental Animal Center at the Second Affiliated Hospital of Fujian Medical University. Mice were housed in a constant temperature (approximately 23 °C) room on a 12-h light–dark cycle. They were allowed free access to food and water. The animal experimental procedures were approved by the Animal Care Committee of the Second Affiliated Hospital of Fujian Medical University, and complied with the “Guidelines for the Care and Use of Laboratory Animals” (No. 2020-268) of the National Institutes of Health.

### Culture and identification of autologous ADSCs

Mouse autologous ADSCs were isolated, cultured, and identified as described in our previously published protocols [27]. Briefly, inguinal adipose tissue was obtained from seven-week-old female C57/BL6 mice, and each mouse was given an ear tag with a specific number. Adipose tissue was washed three times with phosphate-buffered saline (PBS, Meilunbio) to remove blood and then minced into a paste with eye scissors. Then, 0.75 mg/mL collagenase type II (Sigma) was used for enzymatic hydrolysis in a constant temperature shaking bath at 37 °C for 30 min. Next, the digested solution was centrifuged and the cell pellets were re-suspended in DMEM/F12 (1:1) (Hyclone) containing 10% fetal bovine serum (FBS, Gibco) and 1% pen-strep (Gibco); cells were incubated at 37 °C and cultured in a 5% CO2 humidified incubator. The medium was changed every 3 days. The surface markers of the third passage ADSCs were detected by flow cytometry. Cell surface antigens included CD90 (Abcam), CD29 (BioGems), D34 (BioGems), and CD45 (BioGems). ADSCs were treated with commercial osteogenic, chondrogenic, or adipogenic medium (Cyagen) for up to 24 days, following the manufacturer’s instructions. After induction, Oil Red O staining, Alizarin Red staining, and Alcian blue staining (Cyagen) were used to visualize the ability of ADSCs to differentiate into adipocytes, osteoblasts, and chondroblasts. Third passage of autologous ADSCs was used for the subsequent experiments.

### Cell viability assay

The effect of different concentrations of MLT (Sigma) on the viability of ADSCs was detected by the CCK-8 method (Meilunbio). MLT was dissolved in ethanol at a stock concentration of 100 mM and added to the cell culture medium at a final concentration of 1, 5, 10, and 50 µM. ADSCs at 2.5 × 10^5^ cells/well density inoculated in 24-well plates. After 24 h of culture, ADSCs were treated with a gradient concentration of MLT-containing medium for 1, 3, 5, and 7 days, respectively. The MLT-containing medium was refreshed every 24 h. At the end of each time point, ADSCs’ viability was estimated by the CCK-8 assay according to the manufacturer’s protocol. Absorbance values were measured at 450 nm with a microplate reader (TECAN).

### Establishment of POI model and treatment of autologous ADSCs

**Fig. 1:**
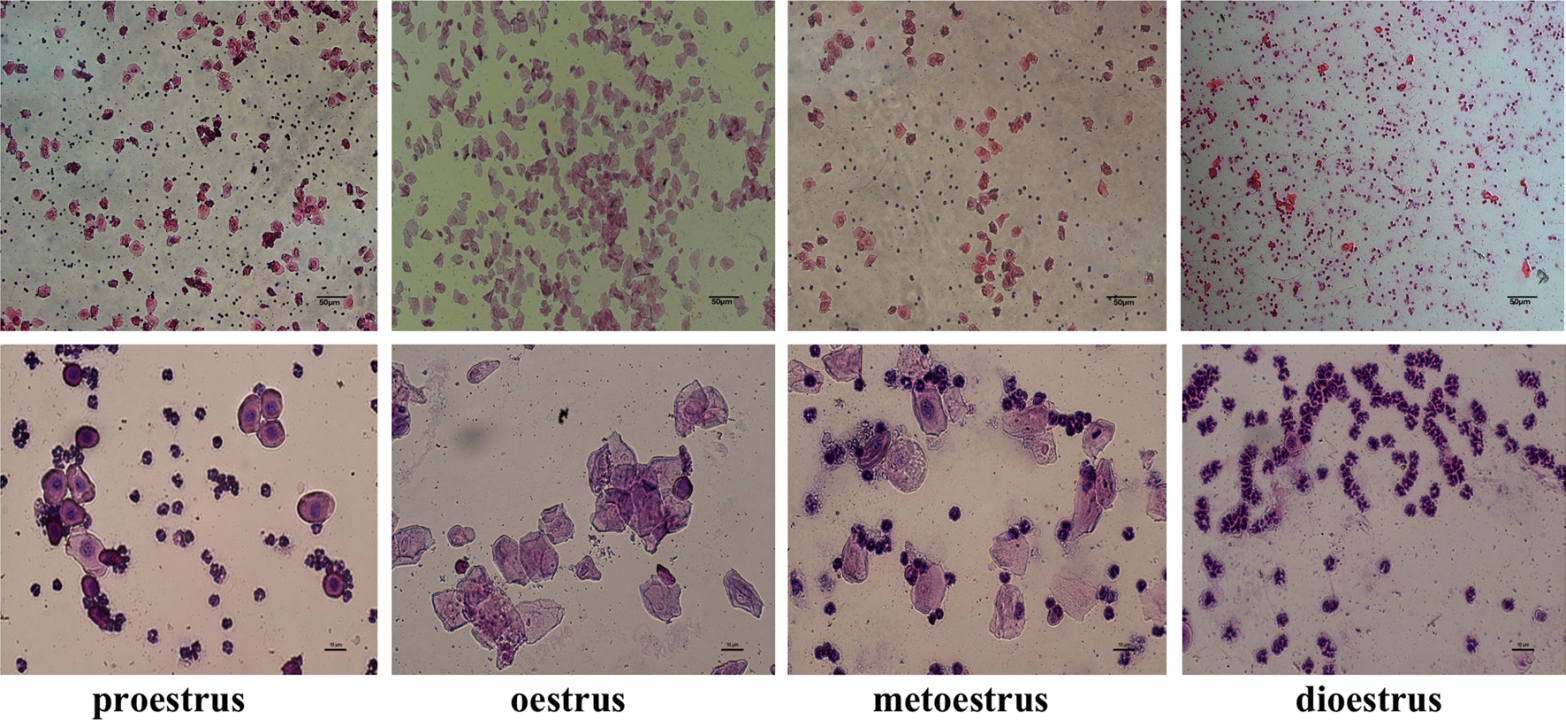
The estrous cycle of mice. Regular estrous cycles include proestrus, oestrus, metoestrus, and dioestrus. Scale bar: 50 or 10 μm

**Fig. 2:**
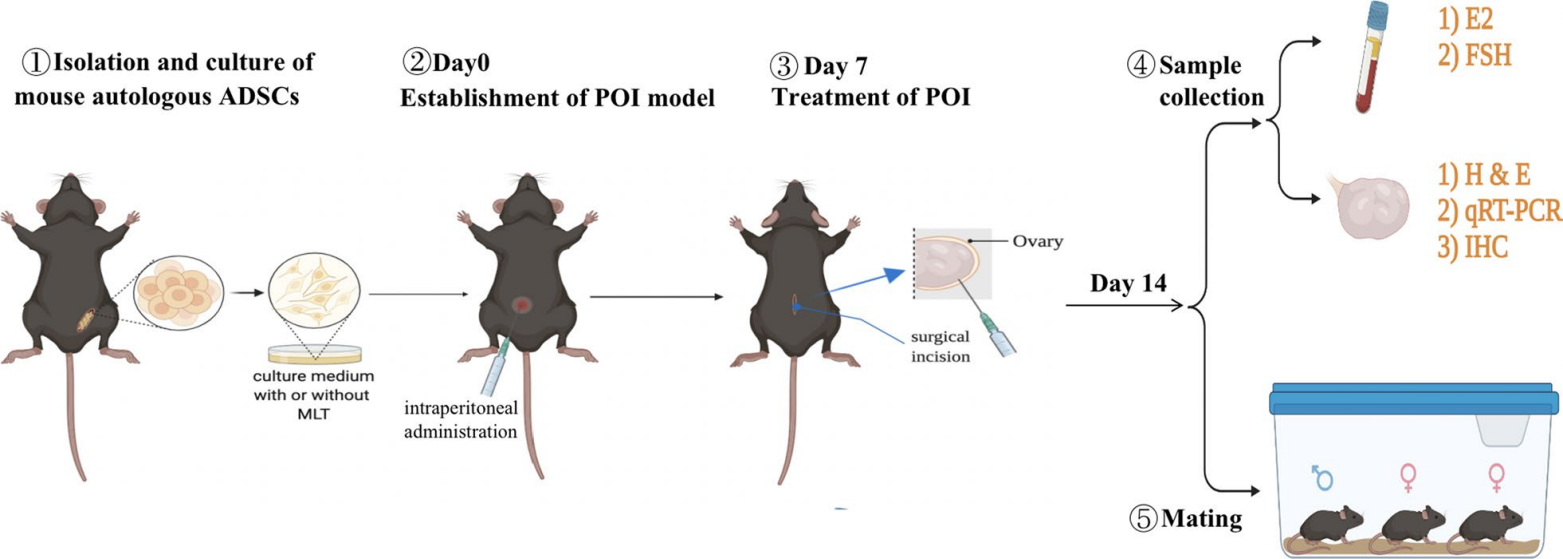
The experimental design. ADSCs, adipose-derived stem cells; POI, premature ovarian insufficiency; MLT, melatonin; E2, estradiol; FSH, follicle-stimulating hormone; AMH, anti-Mullerian hormone; H&E, hematoxylin and eosin; qRT-PCR, quantitative real-time polymerase chain reaction; and IHC, immunohistochemical

The mice were first confirmed to have normal estrus cycles (Fig. 1), and then, the mice were randomly divided into four groups (*n* = 10 each group): control group, PBS group, ADSCs group, and MLT-ADSCs group. According to a previous report [28], mice were intraperitoneally injected with CTX (120 mg/kg, Meilunbio) and busulfan (BUS, 30 mg/kg, Meilunbio) to establish a POI mode. The control group was intraperitoneally injected with an equal volume of 0.9% normal saline. The mice in the PBS group, ADSCs group, and MLT-ADSCs group were first injected with 120 mg/kg CTX and 30 mg/kg BUS to establish the POI model. After 7 days of chemotherapy, the mice in the three groups were anesthetized, and a 1–1.5 cm longitudinal incision was made 1.0 cm below the controversial angle on the dorsal midline of each mouse to expose the bilateral ovaries. Then, mice in the PBS group were injected with 10 μl PBS as a control. For the ADSCs group, 10 μl of PBS containing 1 × 10^6^ autologous ADSCs was sequentially injected into bilateral ovaries according to mouse ear tags [29, 30]. The mice in the MLT-ADSCs group were sequentially injected with 10 μl of PBS containing 1 × 10^6^ autologous MLT-ADSCs into bilateral ovaries, according to the mouse's ear tag. The control group did not receive any treatment. After 7 days of treatment, 20 mice (5 in each group) were killed and peripheral blood serum and bilateral ovaries were collected for subsequent experiments. One ovary was used for hematoxylin and eosin (H&E) and immunohistochemical (IHC) staining, and the other ovary was used for RT-PCR. The remaining 20 mice were continued to be used in fertility experiments. The experimental design is shown in Fig. 2.

### CM-Dil-labeled ADSCs

ADSCs were labeled with CellTracker™ CM-Dil dye (Invitrogen) and tracked following the manufacturer's protocol. ADSCs were seeded at a density of 1.2 × 10^6^ cells/well onto six-well plates. When the cells reached 80% confluency, the MLT-ADSCs group was replaced with an MLT-containing medium. After 72 h, ADSCs and MLT-ADSCs labeled with CM-Dil, respectively. Briefly, CM-Dil was dissolved in ethanol. During the labeling process, cells were suspended in PBS at a concentration of 1 × 10^6^ cells/ml and labeled with 5 µM/ml CM-Dil at 37 °C for 5 min, and incubated at 4 °C for 15 min. After that, the cells were centrifuged at 1000 rpm for 10 min and washed twice with PBS. The cell pellet was re-suspended in DMEM/F12 (1:1) containing 10% FBS and 1% pen-strep. After 48 h labeling, the cells were observed under a fluorescence microscope and then injected into mouse ovaries. Seven days after cell transplantation, the ovaries, hearts, kidneys, and lungs were processed into frozen sections. The sections were incubated with DAPI. Then, all sections were observed under a fluorescence microscope.

### Enzyme-linked immunosorbent assay (ELISA)

Seven days after cell transplantation, four sets of blood samples were collected and centrifuged at 4000 rpm for 10 min. In terms of hormone determination, serum estradiol (E2) and follicle-stimulating hormone (FSH) levels in mice were detected using ELISA kits (Meimian) according to the manufacturer's instructions. Briefly, 10 μl of serum samples were added to each well and incubated at 37 °C for 30 min. Thereafter, wash buffer was used to wash wells five times, which was followed by adding HRP-conjugated reagent to each well and incubation for 30 min at 37 °C. After five additional washes with wash buffer, substrate A and B solutions were added and incubated at 37 °C for 10 min. Finally, 50 μl of stop solution was added, and the optical density was measured at a wavelength of 450 nm with a microplate reader (TECAN). Each sample was repeated three times.

Ovarian dysfunction is related to excessive activation of the dormant primordial follicle, massive apoptosis of granulosa cells, and persistent vascular damage [31, 32]. Previous studies have shown that ADSCs can secrete multiple cytokines, including brain-derived neurotrophic factor (BDNF), hepatocyte growth factor (HGF), vascular endothelial growth factor (VEGF), and nerve growth factor (NGF) [33]. BDNF is now known as an ovarian endocrine factor. It participates in ovarian follicle development [34]. VEGF is essential to promote endothelial cell proliferation and angiogenesis. It also plays a key role in the survival of primordial follicles [35]. Studies have shown that HGF can inhibit apoptosis of granulosa cells and follicle cultures [36]. Therefore, BDNF, VEGF, and HGF were selected to be detected. To detect the paracrine activity of selected cytokines in ADSCs and MLT-ADSCs, ADSCs were seeded at a density of 1.2 × 10^6^ cells/well onto six-well plates. When the cells reached 80% confluency, ADSCs group was replaced with low serum medium (DMEM/F12 containing 1% FBS and 1% pen-strep). MLT-ADSCs group was replaced with low serum MLT-containing medium. After 72 h, the culture supernatant was collected for the subsequent experiments. The levels of VEGF, HGF, and BDNF were measured by Mouse VEGF ELISA Kit (MultiSciences Biotech), Mouse BDNF ELISA Kit (MultiSciences Biotech), and Mouse HGF ELISA Kit (Jianglaibio), according to the manufacturer’s protocol. Each sample was repeated three times.

### H&E staining and ovarian follicle count

Ovaries were fixed with 4% paraformaldehyde for 24 h, embedded in paraffin, and serially sectioned (5 µm), followed by H&E staining (Solarbio). Three sections were randomly selected from each ovary. Follicles were classified as primordial, primary, secondary, and atretic as previously described [37, 38]. Different categories of follicles were counted under light microscopy. To avoid recounting, only follicles containing oocytes were counted.

### Quantitative real-time polymerase chain reaction (qRT-PCR)

Total RNA was extracted from ovarian tissue using the RNAeasy™ Animal RNA Isolation Kit with Spin Column (Beyotime). Purity and concentration of RNA samples were quantified with a spectrophotometer. Reverse transcription reactions and cDNA synthesis were performed using the PrimeScript™ RT Reagent Kit with gDNA Eraser (Perfect Real Time) (Takara), following the manufacturer's protocol. SIRT6, NF-κB, and BDNF mRNA expression levels were quantified by qRT-PCR with TB Green® Premix Ex Taq™ (TliRNaseH Plus) (Takara). The primer sequences are listed in Table 1. The expression of β-actin was used as an internal reference. Each group included five samples, and each sample was repeated in triplicate. The 2^−△△CT^ method was performed to calculate relative gene expression levels.

**Table 1:** qRT-PCR primers sequences

Primers	Sequences 5′-3′
SIRT6	F: 5′- GCTGAGGGACACCATCCTAGA -3′
	R: 5′- GTAGCCAGCGGCAGGTTC -3′
NF-κB	F: 5′- GTGGGGACTACGACCTGAATG -3′
	R: 5′- CTGCACCTTGTCACACAGTAGG -3′
BDNF	F: 5′- CGACGACATCACTGGCTGACAC -3′
	R: 5′- GAGGCTCCAAAGGCACTTGACTG -3′
β-actin	F: 5′- CCTAAGGCCAACCGTGAAAAG -3′
	R: 5′- AGGCATACAGGGACAGCACAG -3′

SIRT6, sirtuin 6; NF-κB, nuclear factor kappa B; BDNF, brain-derived neurotrophic factor; F, forward; and R, reverse

### IHC staining

After serially sectioning, deparaffinization, rehydration, and antigen retrieval, the ovarian sections were placed in 3% hydrogen peroxide to block endogenous peroxidase activity, followed by blocking with goat serum for 1 h. Thereafter, sections were incubated overnight at 4 °C with primary antibodies against SIRT6 (HUABIO, 1:200), NF-κB (Abcam, 1:2000), and BDNF (HUABIO, 1:200). After rinsing, the sections were incubated for 60 min at room temperature with appropriate secondary antibodies. Then, slides were stained with 3, 3-diaminobenzidine and counterstained with hematoxylin. Finally, immunoreactivity was quantified by Image Pro Plus 6.0, and three to five fields on each slide were randomly selected for determining the mean optical density.

### Fertility experiments

Twenty mice (*n* = 5 per group) were randomly selected for the mating experiment. Seven days after cell transplantation, female mice were mated with sexually mature male mice in a 2:1 ratio for 24 days. The presence of a vaginal plugs was defined as successful mating. After each pregnancy, males were randomly rotated among cages and the number of offspring per female was recorded.

### Statistical analysis

Drawing graph and statistical analyses were performed using the GraphPad Prism version 9.3 (GraphPad Software Inc., La Jolla, CA). Unpaired Student’s t tests were used for comparisons between two groups. Multiple comparison among three or more groups was evaluated using one-way analysis of variance (ANOVA). Significance values are expressed as: ns = not significant, *p < 0.05, **p < 0.01, ***p < 0.001, and ****p < 0.0001.

## Results

### Phenotypic characteristics and differentiation of autologous ADSCs

**Fig. 3:**
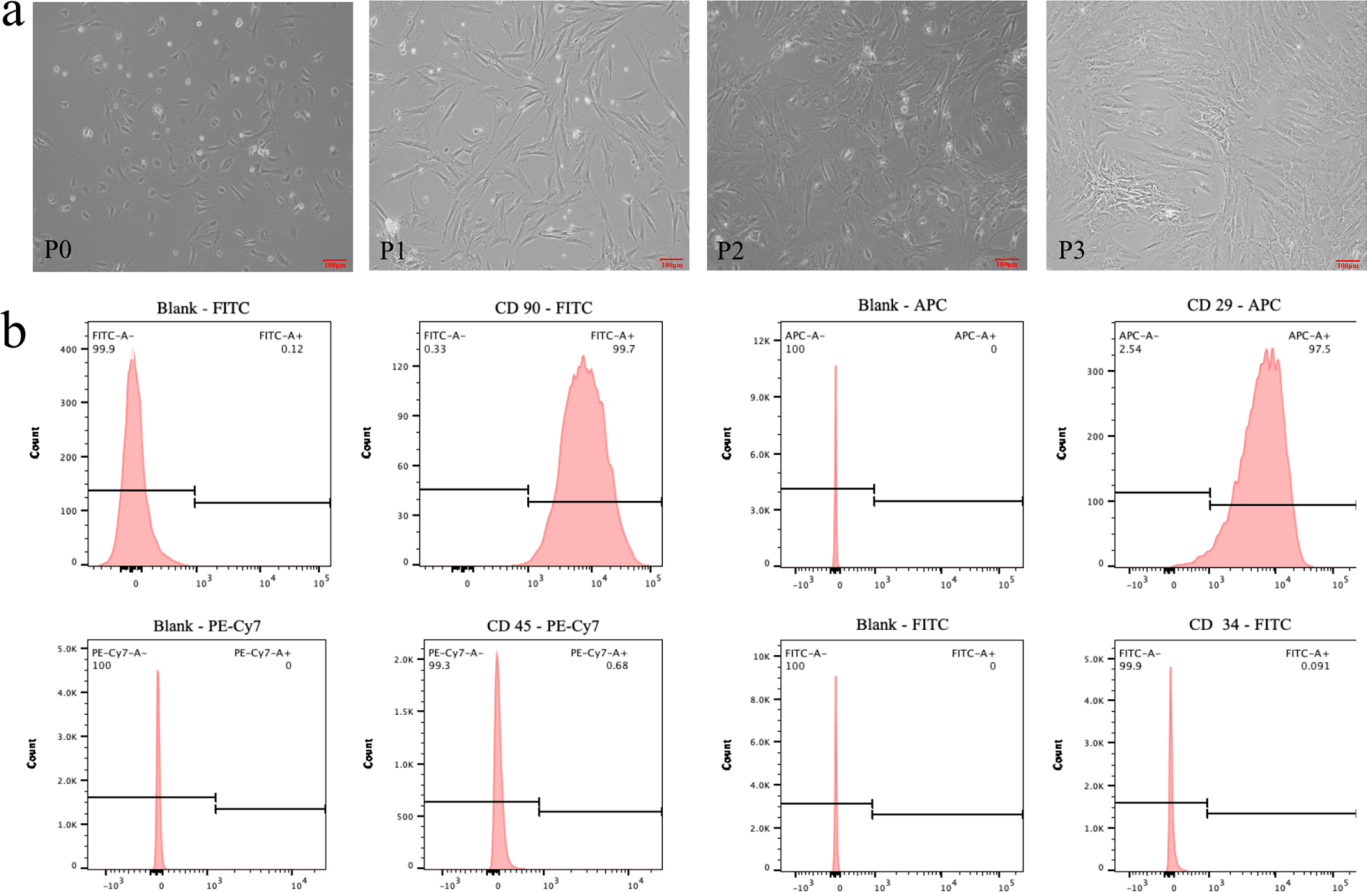
The morphology and characterization of autologous ADSCs. **a** The fibroblastoid shape of ADSCs from passage 0 (P0) to P3 (scale bar: 100 μm). **b** Flow cytometry analysis of cells surface markers in ADSCs. CD 90: 99.7%; CD 29: 97.5%; CD 45: 0.68%; and CD 34: 0.091%

**Fig. 4:**
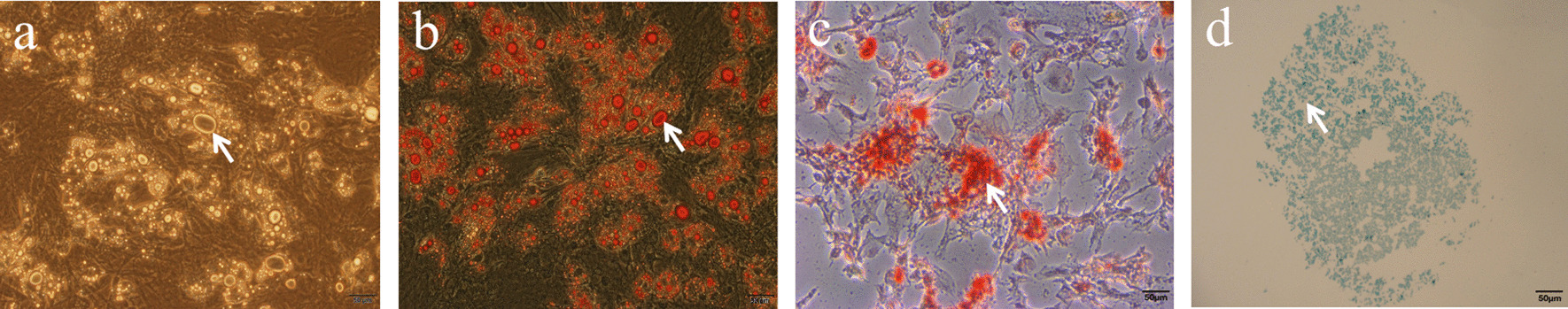
The differentiation of autologous ADSCs. **a** Lipid droplets secreted by differentiated adipocytes were observed under bright-field microscope. The arrow indicates lipid droplets. **b**–**d** The adipogenic, osteogenic, and chondrogenic differentiation of ADSCs was assessed by Oil Red O staining, Alizarin Red staining, and Alcian blue staining, respectively. The arrows in b-d indicate lipid droplets, calcium nodules, and mucopolysaccharides in chondrocytes, respectively. Scale bar: 50 μm

Freshly isolated and cultured autologous ADSCs were adherent and most of them had a polygonal morphology. Adherent cells can expand in vitro and gradually came to resemble spindle-shaped fibroblast-like cells (Fig. 3a). By the third passage, ADSCs surface markers were detected by flow cytometry. The results showed that ADSCs were positive for CD90 and CD29 but negative for CD45 and CD34 (Fig. 3b). Moreover, these cells showed the ability to differentiate into adipocytes, osteoblasts, and chondroblasts in vitro (Fig. 4).

### Melatonin promotes the proliferation of ADSCs in vitro

**Fig. 5:**
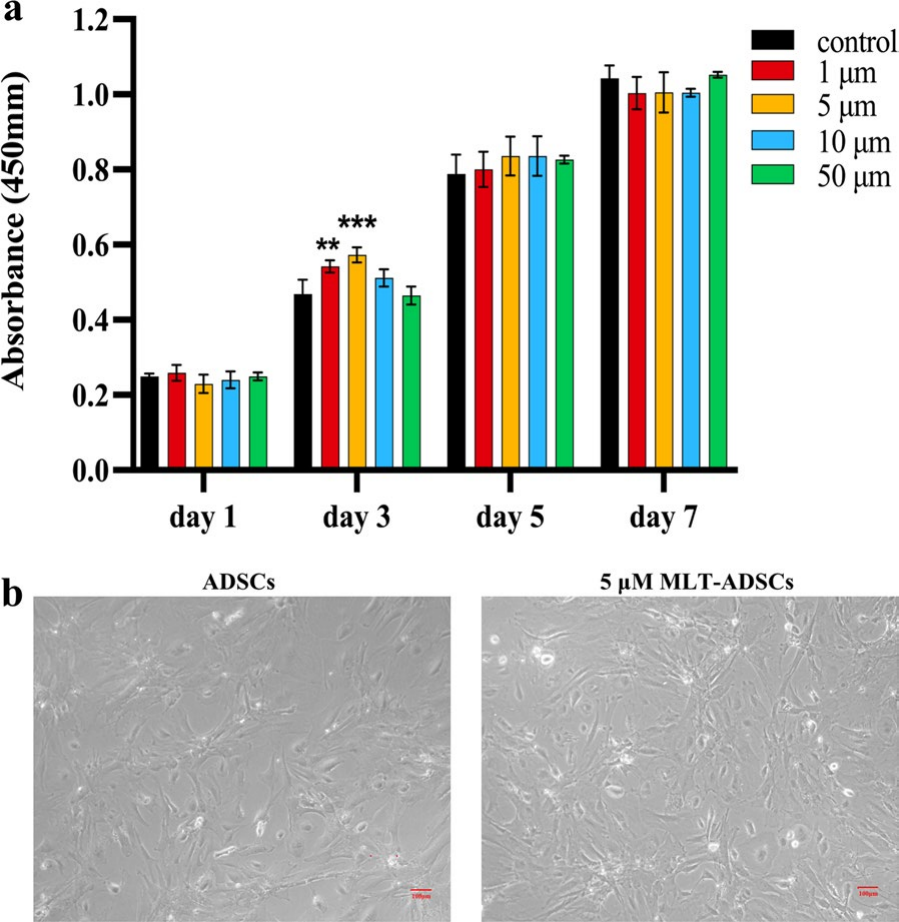
Assay of the activity of ADSCs treated with MLT. **a** Dose-dependent and time-course tests to detect the optimal concentration of MLT for promoting ADSCs proliferation. The cell proliferation levels were determined by cck-8 assay on days 1, 3, 5, and 7. (**p < 0.01, and ***p < 0.001 vs. control). **b** ADSCs morphology with or without 5 μM MLT. Scale bar: 50 μm

The optimal concentration of MLT to promote the proliferation of ADSCs was selected by the CCK-8 assay (Fig. 5a). The results revealed the absorbance (an indicator of cell proliferation) on day 3 was significantly higher than that of the control group when MLT concentration was 1 µM (*p* = 0.0034) and 5 µM (*p* = 0.0001). However, compared with the control group, the effect of MLT on cell proliferation did not increase significantly on days 1 and 5 and even inhibited the proliferation of ADSCs on day 7.

Taken together, we selected 5 µM MLT to pretreat ADSCs for 3 days for subsequent studies. As anticipated, 5 µM MLT treatment for 3 days increased the number of the ADSCs and did not change cell morphology compared to the normal cultured ADSCs (Fig. 5b).

### Melatonin promotes ADSCs to secrete cytokines

**Fig. 6:**
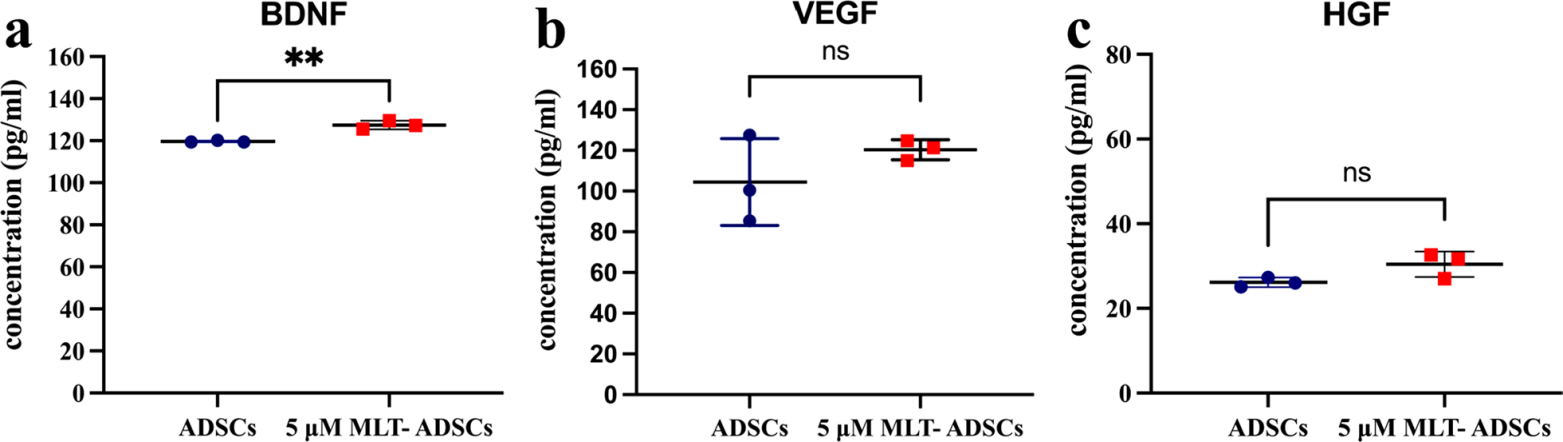
The levels of BDNF, VEGF, and HGF were measured by ELISA assay in ADSCs and 5 μM MLT-ADSCs (ns = not significant, **p < 0.01 vs. ADSCs groups). **a** The levels of BDNF in ADSCs and 5 μM MLT-ADSCs. **b** The levels of VEGF in ADSCs and 5 μM MLT-ADSCs. **c** The levels of HGF in ADSCs and 5 μM MLT-ADSCs. BDNF, brain-derived neurotrophic factor; VEGF, vascular endothelial growth factor; and HGF, hepatocyte growth factor

Accumulating studies have reported that therapeutic effect of MSCs is related to the formation of a secretome [39]. Therefore, we further explored whether MLT could enhance the paracrine capacity of ADSCs by ELISA assay. The results showed that MLT treatment significantly up-regulated the expression of BDNF (*p* = 0.0028, Fig. 6a). VEGF and HGF also increased, but the difference was not statistically significant (*p* = 0.2784 and 0.0801, respectively, Fig. 6b–c).

### Melatonin-pretreated autologous ADSCs rescued ovarian function in POI mice

**Fig. 7:**
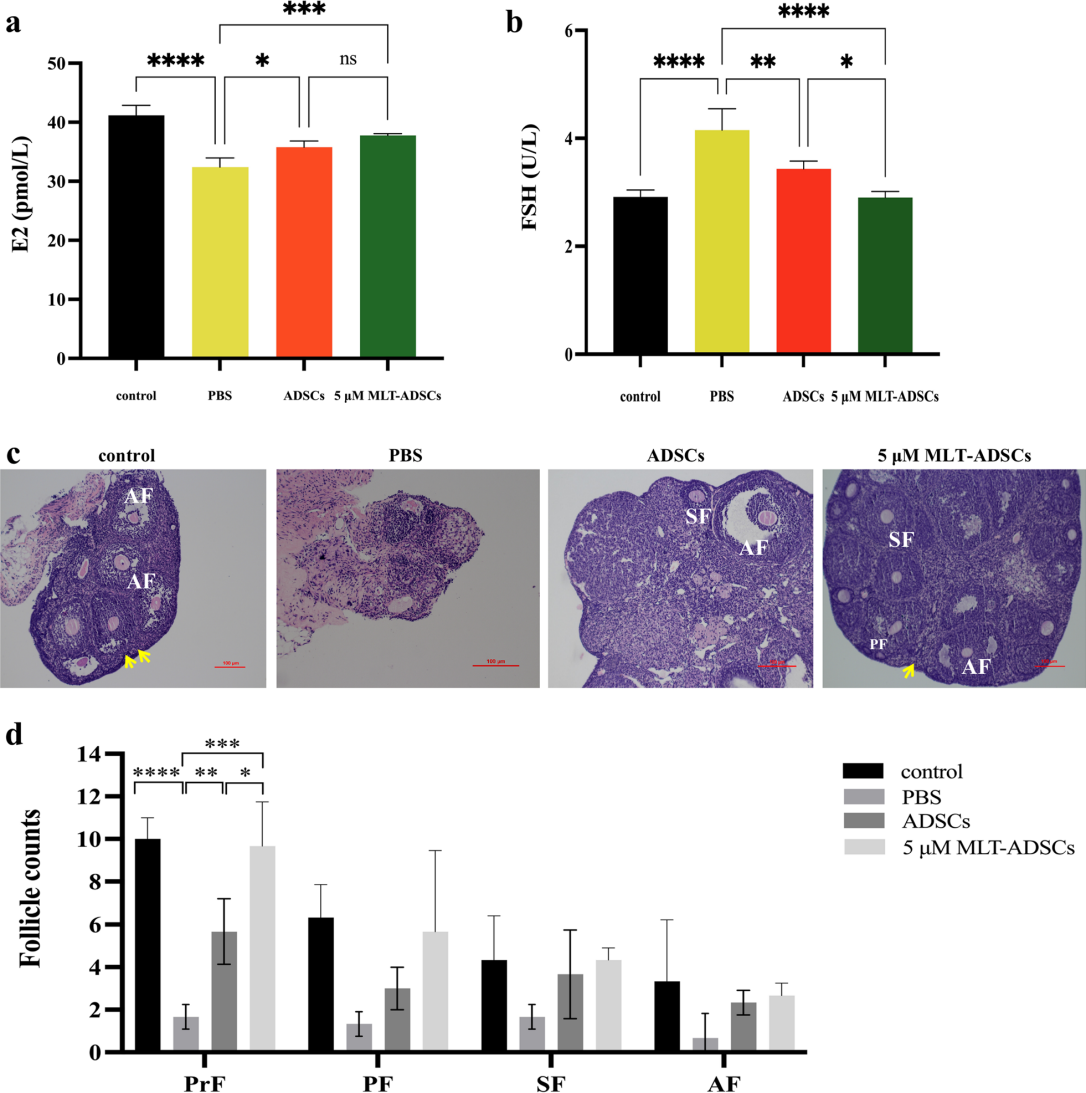
Effects of ADSCs and 5 μM MLT-ADSCs transplantation in mice with POI. **a** Changes in serum E2 levels. **b** Changes in serum FSH levels. **c** Ovarian histological changes were analyzed using H&E staining. Scale bar: 100 μm. Yellow arrows indicate PrFs located in the ovarian cortex. **d** The number of follicles at different stages was counted and compared in the control, PBS, ADSCs, and 5 μM MLT-ADSCs groups. PrF, primordial follicle; PF, primary follicle; SF, secondary follicle; AF, antral follicle; E2, estradiol; and FSH, follicle-stimulating hormone. (ns = not significant, *p < 0.05, **p < 0.01, ***p < 0.001 and ****p < 0.0001)

First, PBS, 1 × 10^6^ autologous 5 µM MLT-ADSCs, or 1 × 10^6^ autologous ADSCs were injected into ovaries of POI mice to elucidate the therapeutic effect. The ELISA results showed that compared with the control group, the E2 level (*p* < 0.0001, Fig. 7a) in the PBS group was significantly decreased, and the FSH level (*p* < 0.0001, Fig. 7b) was notably elevated. After 7 days of treatment with ADSCs or 5 µM MLT-ADSCs, E2 (ADSCs group, *p* = 0.0128; 5 µM MLT-ADSCs group, *p* = 0.0003, Fig. 7a) had significantly higher levels, while FSH (ADSCs group, *p* < 0.0039; 5 µM MLT-ADSCs group, *p* < 0.0001, Fig. 7b) was significantly lower compared to the PBS group. Meanwhile, compared with ADSCs group, the 5 µM MLT-ADSCs group had better FSH recovery effect (*p* = 0.0282, Fig. 7b).

Next, H&E staining revealed that CTX and BUS administration resulted in ovarian tissue atrophy, follicle loss, and vascular damage in the POI group. Compared with the control group, the number of primordial follicles (PrFs) was significantly decreased in the POI group (*p* < 0.0001, Fig. 7d), while the number of primary follicles (PF), secondary follicles (SF), and antral follicles (AF) was not significantly decreased (Fig. 7d). The amount of PrF was significantly increased after ADSCs and 5 µM MLT-ADSCs treatment compared with the PBS group (ADSCs group, *p* = 0.0051; 5 µM MLT-ADSCs group, *p* < 0.0001, Fig. 7d). Furthermore, the amount of PrF was significantly increased in the 5 µM MLT-ADSCs group compared with the ADSCs group (*p* = 0.0224, Fig. 7d). In addition, the number of PF, SF, and AF also showed an increasing trend, but there was no statistical difference with the PBS group (Fig. 7d).

**Fig. 8:**
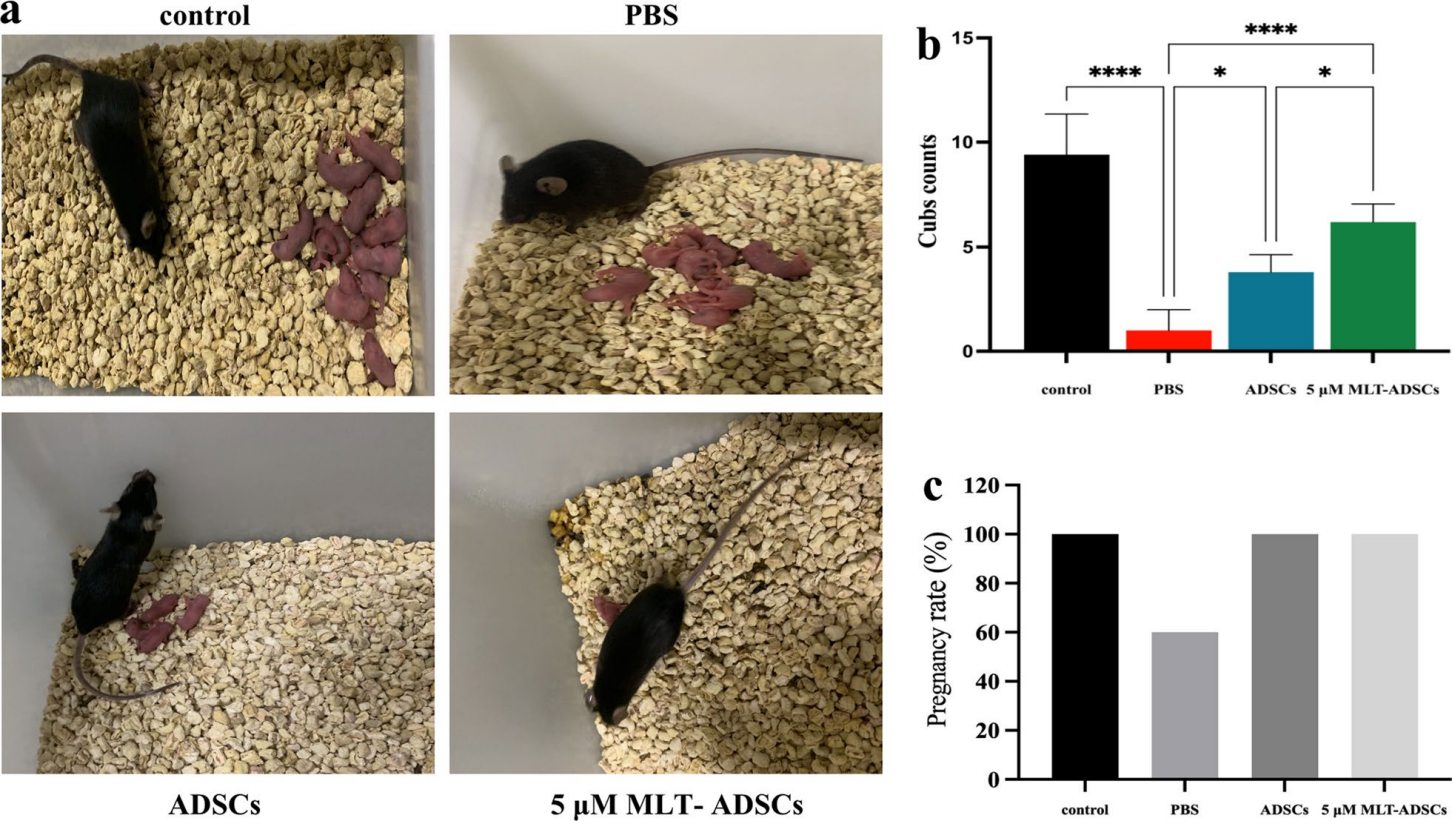
Results of the fertility experiment (*p < 0.05 and ****p < 0.0001) **a** Reproductive outcomes. **b** The cubs counts of per group. **c** The pregnancy rate of per group

Furthermore, we examined the reproductive capacity of the transplanted mice. The results of mating trials showed, compared with the control group (47 pups), that chemotherapy significantly decreased the offspring numbers of mice in the PBS group (5 pups) (*p* < 0.0001, Fig. 8b). Similarly, the pregnancy rate of mice in the POI group was lower than that in the control group (Fig. 8c). After ADSCs or 5 µM MLT-ADSCs treatment, the total litter size of the treated POI mice was significantly higher than those in the PBS group (ADSCs group, *p* = 0.0126; 5 µM MLT-ADSCs group, *p* < 0.0001, Fig. 8b); there were a total 19 pups in mice transplanted with ADSCs and 31 pups in mice that received 5 µM MLT-ADSCs (*p* = 0.0348, Fig. 8b). The pregnancy rate of mice in the ADSCs and 5 µM MLT-ADSCs group was higher than those in the PBS group (Fig. 8c). In addition, no significant abnormalities were observed in any offspring seven days after birth. The results showed that ADSCs transplantation partially restored ovarian functions in POI mice, while 5 µM MLT-ADSCs enhanced this effect.

**Fig. 9:**
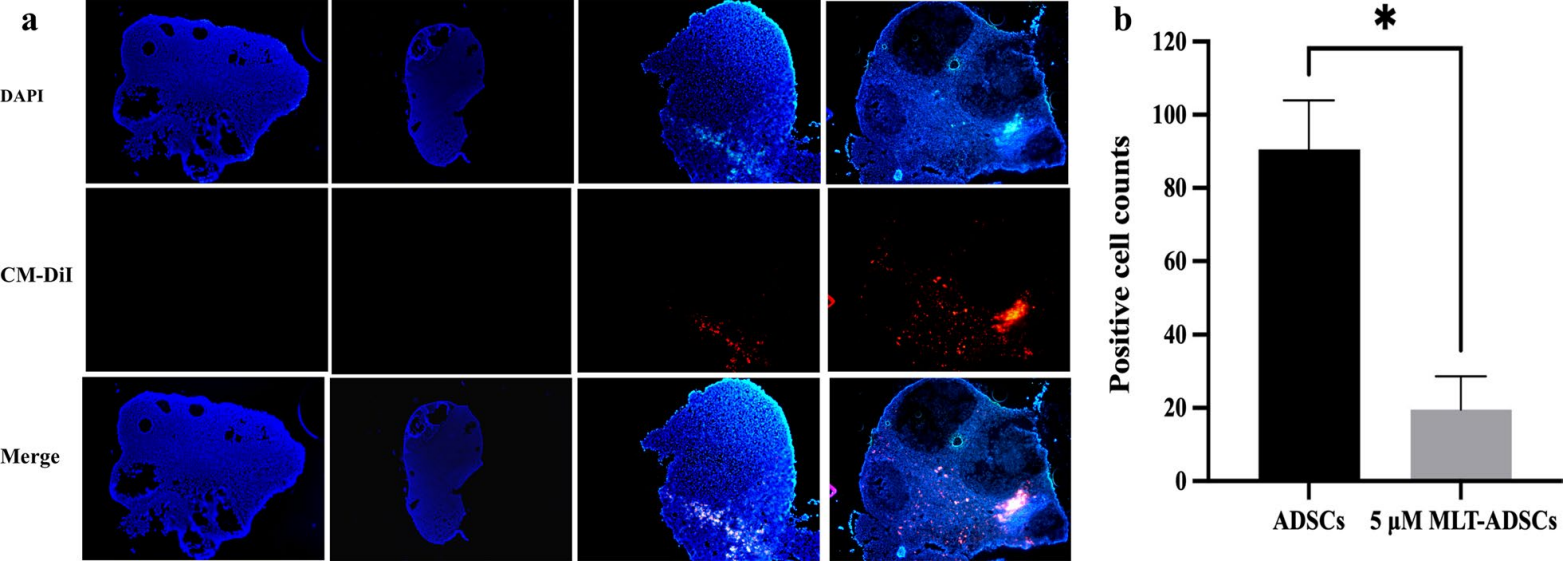
In vivo tracking after cell transplantation. **a** Fluorescence microscopy analysis of ovarian tissue sections. Scale bar: 100 μm. **b** The number of positive cells was counted and compared in the ADSCs and 5 μM MLT-ADSCs groups (*p < 0.05)

**Fig. 10:**
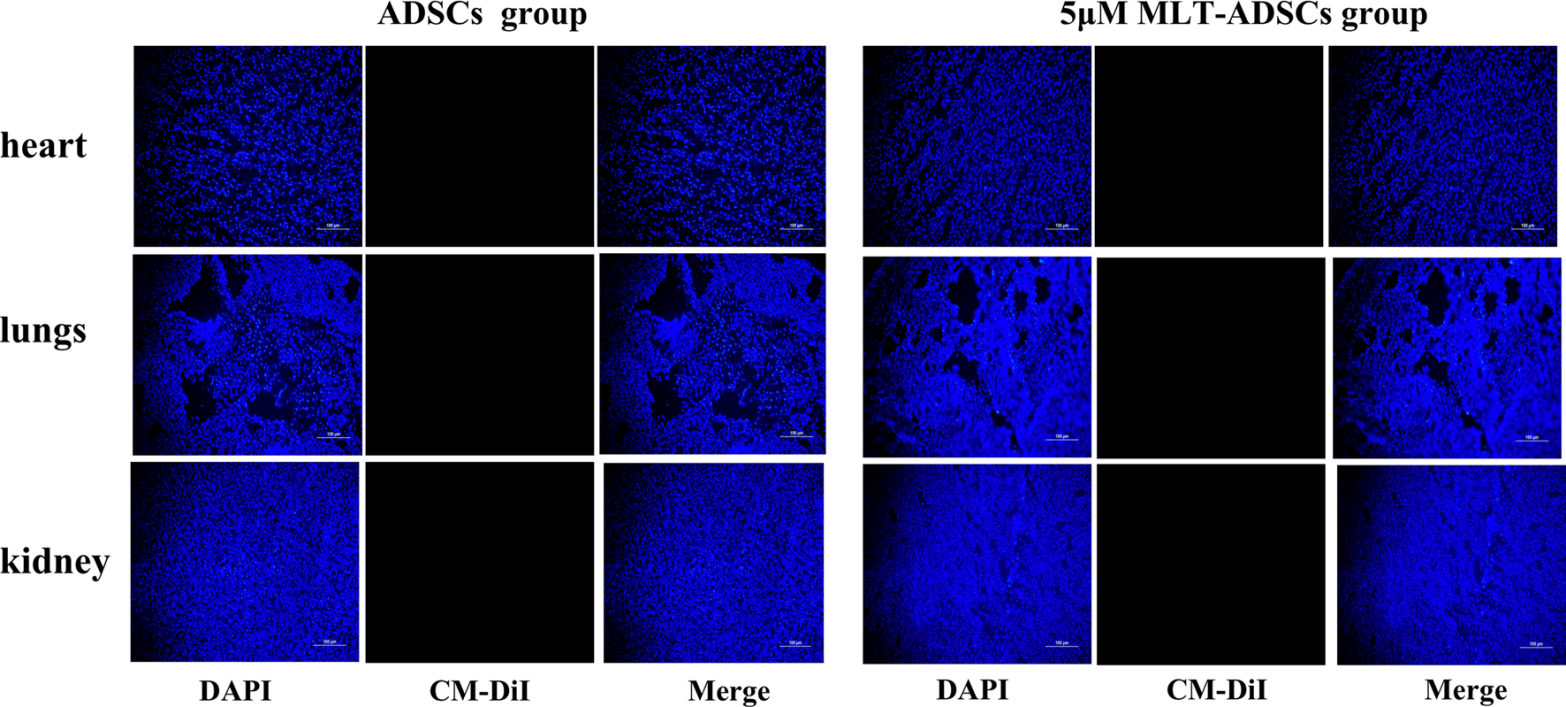
In vivo tracking after cell transplantation. Fluorescence microscopy analysis of heart, lungs, and kidney sections from ADSCs and 5 μM MLT-ADSCs groups. Scale bar: 100 μm

Finally, we pre-labeled CM-Dil on ADSCs and 5 µM MLT-AMSCs, tracked the transplanted cells in vivo, and found CM-Dil-positive cells showed red fluorescence. As expected, we did not observe any CM-Dil-positive cells in the ovarian tissue sections of the control and PBS groups. However, we observed CM-Dil-positive cells in both ADSCs and 5 µM MLT-ADSCs groups in ovarian tissue sections (Fig. 9a). Quantitation of the number of positive cells in the ovaries revealed that the number of CM-Dil-positive cells in the ovarian tissue sections was higher in the 5 µM MLT-ADSCs group than in the ADSCs group (Fig. 9b). A red fluorescent signal was not observed in the heart, kidney, and lung of the ADSCs group and 5 µM MLT-AMSCs groups (Fig. 10). These results demonstrated that the transplanted ADSCs were mainly recruited and located in the ovaries, which is one of the most important target organs of chemotherapy injury [40, 41]. Moreover, 5 µM MLT-pretreated ADSCs exhibited enhanced cell retention in ovaries.

### Melatonin-pretreated autologous ADSCs enhance the SIRT6/NF-κB signaling pathway

**Fig. 11:**
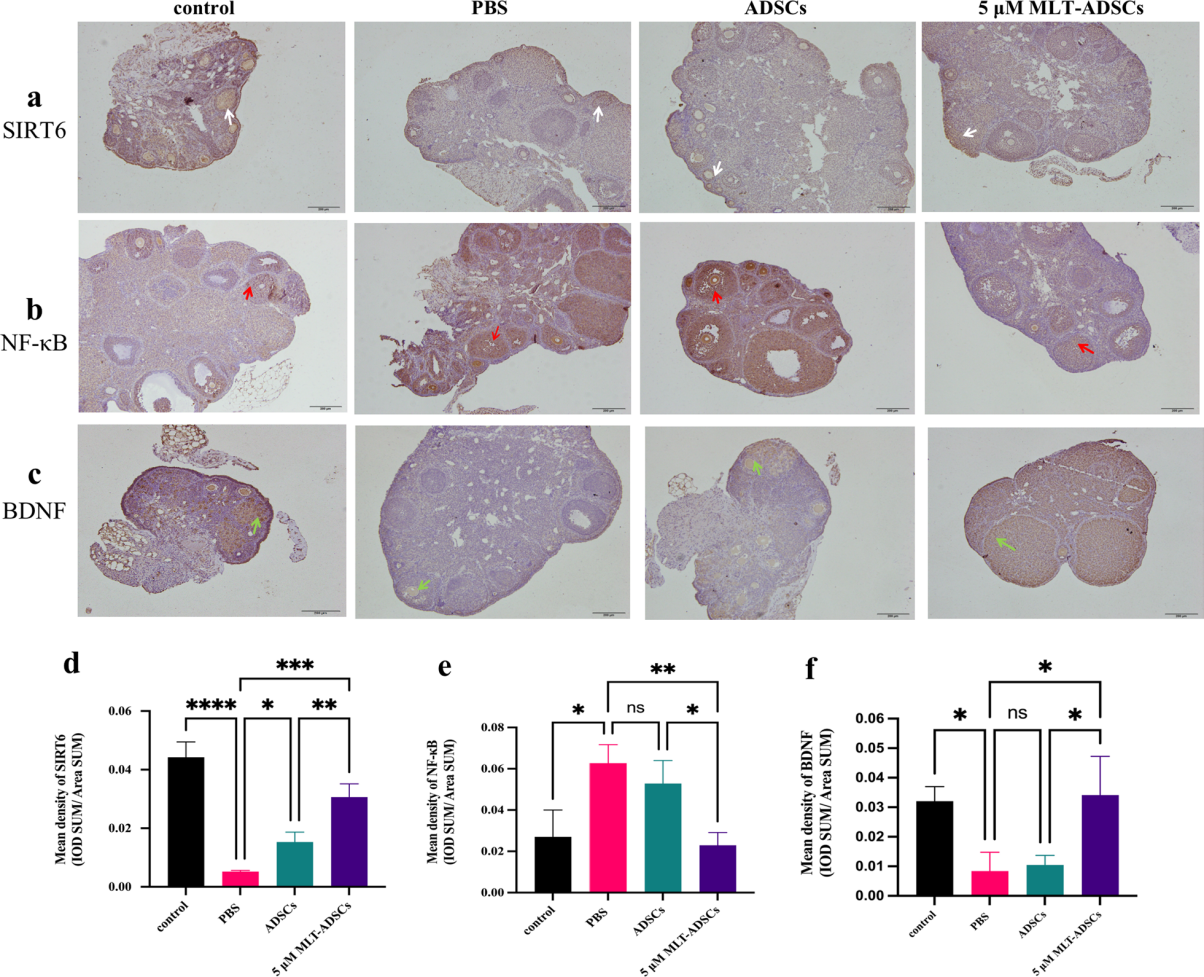
The expression levels of SIRT6, NF-κB, and BDNF in the ovaries were detected using immunohistochemical staining. **a**–**c** A brown-yellow color indicates positive expression of the target protein. The white arrow represents SIRT6 expression, the red arrow represents NF-κB expression, and the green arrow represents BDNF expression. Scale bar: 200 μm. **d**–**f** The statistical charts of the three kinds of protein expression in the four groups. SIRT6, sirtuin 6; NF-κB, nuclear factor kappa B; and BDNF, brain-derived neurotrophic factor (ns = not significant, *p < 0.05, **p < 0.01, ***p < 0.001, ****p < 0.001)

To investigate whether the SIRT6/NF-κB signaling pathway was involved in ovarian repair in POI mice, IHC and qRT-PCR were used to detect the expressions of SIRT6 and NF-κB in ovarian tissues of mice in each group. The results of IHC showed that compared with the control group, the level of SIRT6 was significantly decreased in the PBS group (*p* < 0.0001, Fig. 11d), and the level of NF-κB was significantly increased (*p* = 0.0110, Fig. 11e). The staining intensity of SIRT6 in the ADSCs (*p* = 0.0487, Fig. 11d) and 5 µM MLT-ADSCs groups (*p* = 0.0002, Fig. 11d) was higher than that in the PBS group. As shown in Fig. 11e, the level of NF-κB in the 5 µM MLT-ADSCs group was significantly lower than that in the POI group (*p* = 0.0060). The staining intensity of NF-κB in the ADSCs group was lower than that in the PBS group, but the difference was not statistically significant (*p* = 0.6500, Fig. 11e). Moreover, compared with the ADSCs group, the 5 µM MLT-ADSCs group had significantly higher SIRT6 expression (*p* = 0.0053, Fig. 11d) and significantly lower NF-κB expression (P = 0.0285, Fig. 11e).

**Fig. 12:**
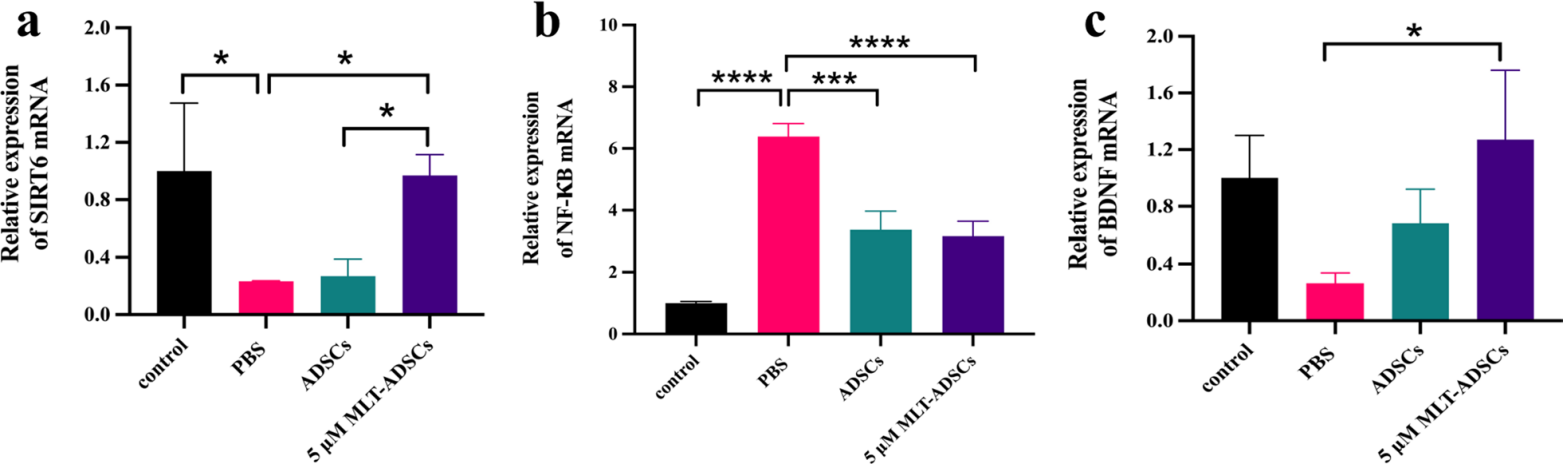
qRT-PCR results. **a** Relative expression of SIRT6 mRNA in four groups. **b** Relative expression of NF-κB mRNA in four groups. **c** Relative expression of BDNF mRNA in four groups. SIRT6, sirtuin 6; NF-κB, nuclear factor kappa B; and BDNF, brain-derived neurotrophic factor (ns = not significant, *p < 0.05, **p < 0.01, ****p < 0.001)

Similarly, qRT-PCR results showed that POI mice had significantly lower SIRT6 expression (*p* = 0.0254, Fig. 12a) and significantly higher NF-κB expression (*p* < 0.0001, Fig. 12b) compared with controls. After ADSCs treatment, the expression of SIRT6 was slightly higher than in the PBS group, but the difference was not statistically significant (*p* = 0.9978, Fig. 12a). SIRT6 expression was significantly increased in the 5 µM MLT-ADSCs group compared with the PBS group (*p* = 0.0311, Fig. 12a). The SIRT6 mRNA expression in the 5 µM MLT-ADSCs group was significantly higher than in the ADSCs group. In addition, NF-κB expression was decreased in both the ADSCs group (*p* = 0.0001, Fig. 12b) and the 5 µM MLT-ADSCs group (*p* < 0.0001, Fig. 12b), and the difference was statistically significant. It suggested that SIRT6/NF-κB signaling pathway was involved in the recovery of ovarian function induced by transplantation of ADSCs and 5 µM MLT-ADSCs.

Furthermore, the above experiments showed that MLT promoted the secretion of BDNF from ADSCs, and we further detected the expression of BDNF in ovarian tissues by IHC staining and qRT-PCR to confirm the effect of MLT. The results of IHC staining showed that the expression of BDNF in the ADSCs group was slightly higher than in the PBS group, but the difference was not statistically significant (*p* = 0.9877, Fig. 11f). The expression of BDNF in the 5 µM MLT-ADSCs group was significantly higher than that in the POI group (*p* = 0.0169, Fig. 11f). Meanwhile, the expression of BDNF in the 5 µM MLT-ADSCs group was also significantly higher than that in the ADSCs group (*p* = 0.0262, Fig. 11f). However, qRT-PCR results showed that only the 5 µM MLT-ADSCs group had significantly higher BDNF mRNA expression than the PBS group (*p* = 0.0183, Fig. 12c).

## Discussion

In this study, our data demonstrate that (1) as a natural hormone without major side effects, MLT can promote ADSCs proliferation and enhance their secretory function in a concentration- and time-dependent manner; (2) MLT-primed ADSCs transplantation can significantly improve chemotherapy-induced ovarian injury in mice, including partially restoring hormones levels and increasing primordial follicle; and (3) MLT-primed ADSCs transplantation may be through the SIRT6/NF-κB signaling pathway to promote ovarian repair, which require further research in the future.

In the past few years, MSCs have made significant progress in their application in regenerative medicine, including the treatment of POI [42]. However, MSCs therapy has many limitations, including insufficient cell sources, immunogenicity, cellular replicative senescence, and ethical controversies. ADSCs have a wide range of sources and are easy to isolate, making them ideal therapeutic cells. A large number of ADSCs in adipose tissue can reduce long-term subculture and escape the risk of chromosome abnormalities. Moreover, ADSCs can realize autologous transplantation and maintain genetic stability, which is more in line with ethical requirements. Therefore, our study uses autologous ADSCs to treat POI mice. Nevertheless, the therapeutic effect of MSCs is still affected by various unfavorable factors such as oxidative stress, chronic inflammation, and an ischemic microenvironment in vivo [43]. Previous studies have shown that MLT can increase expression of antioxidative enzymes and mitogenic factors in MSCs and restore cell adhesion and apoptosis damaged by hydrogen peroxide [44]. Luchetti et al. reported that MLT could control the survival and differentiation of MSCs by regulating the Wnt/β-catenin pathway, MAPKs, and TGF-β signaling pathways [45]. Furthermore, a recent study found that MLT could activate NF2 and inhibit endoplasmic reticulum stress, which in turn significantly reduced the aging of ADSCs [19]. Therefore, in this study, MLT was used as an additive to culture ADSCs in vitro*.* Our study performed dose-dependent and time-course tests to detect the optimal concentration and timing of MLT pretreatment. The results of CCK-8 showed that the proliferation rate of ADSCs was significantly increased after 5 µM MLT treatment for 3 days. Intriguingly, MLT also increased the cytokines secreted by ADSCs, which help to inhibit granulosa cell apoptosis and stimulate angiogenesis and follicle growth and development. In our results, only BDNF was significantly up-regulated after MLT pretreatment. Moreover, the IHC and qRT-PCR results showed that the expression of BDNF in the ovaries of the 5 µM MLT-ADSCs group was remarkably higher than in the PBS group. VEGF and HGF showed an upward trend, but neither of them reached statistical significance. This may be due to the different mechanism of MLT signaling in ADSCs, which needs to be further explored.

Intravenous injection is a common modality for MSCs transplantation, but this route of treatment results in most MSCs remaining in the lung and spleen [46]. Growing evidence suggests that direct injection of cells into target tissues improves their therapeutic efficacy [47]; therefore, we used intraovarian injection of autologous 5 µM MLT-ADSCs to treat POI. The first indication that transplantation of 5 µM MLT-ADSCs reduced the deleterious effects of chemotherapy on the ovaries was that the size of the organ had almost returned to normal. In addition, the effect of 5 µM MLT-ADSCs on ovarian function was confirmed by determination of hormone levels, ovarian histological inspection, and the mating trail. In our study, the E2 level and litter size of the 5 µM MLT-ADSCs group and ADSCs group recovered to varying degrees, the FSH level continued to decline, and the number of follicles increased, especially primordial follicles. Importantly, our data suggest the recovery of ovarian function in the 5 µM MLT-ADSCs group was superior to that in the ADSCs group. In this study, we use CM-Dil to label ADSCs and selected day 7 after cell transplantation as a time point to detect fluorescence expression to verify the survival rate of ADSCs after transplantation. Our results are consistent with previous studies that CM-Dil-positive cells were recruited and located to the ovarian interstitium but not to the follicle [48]. We believe that the paracrine effect of ADSCs plays a major role in the improvement of ovarian structure and function, while direct regeneration of ADSCs plays a lesser role. Unfortunately, we do not have enough evidence to prove this. More detailed studies are needed to figure out whether ADSCs transplantation constitutes regenerative cell therapy or cell-based cytokine therapy.

In recent years, chemotherapy-induced POI has attracted extensive attention. CTX is still considered to be one of the most gonadotoxic drugs, which accelerates PrF activation and induces granulosa cell apoptosis and follicular atresia [31]. Similarly, BUS can also cause ovarian injury because of its cytotoxic effects [49]. Furthermore, the occurrence of POI is associated with inflammation, which affects oocytes through follicle microenvironment, oxidative stress, and granulocyte–macrophage colony-stimulating factor [20]. It has been shown that MSCs reverse ovarian aging owing to their homing ability and immunomodulatory and paracrine effects [50, 51], but little is known about the pathways involved. Studies have confirmed that SIRT6 protein expressions levels are positively correlated with the size of the primordial follicle pool, suggesting that they may be a potential marker for clinical assessment of ovarian reserve [52]. On the contrary, NF-κB promotes the progression of POI by regulating a variety of proinflammatory factors, such as tumor necrosis factor-α, interleukin-1β (IL-1β), IL-2, and IL-6 [53]. Therefore, inhibiting the expression of NF-κB may help to reduce the degree of ovarian damage. In fact, the literature reports that SIRT6 is negatively correlated with NF-κB [26]. In this study, the POI mouse model was established by intraperitoneal injection of CTX and BUS. We found that, compared with the PBS group, both SIRT6 protein and mRNA levels were increased in the 5 µM MLT-ADSCs group, whereas NF-κB levels decreased. Compared with the PBS group, the levels of SIRT6 increased in the ADSCs group, while NF-κB decreased slightly, but the difference was not statistically significant. Our study shows for the first time that MLT-ADSCs can treat POI. However, how MLT-ADSCs activate the SIRT6/NF-κB pathway is still unclear, and this issue requires further investigation.

We outlined the next few steps in our ongoing study to further elucidate the mechanism of autologous MLT-ADSCs in the treatment of POI. First, we will further study the regulatory role of the SIRT6/NF-κB pathway in ADSCs therapy for POI. Second, because of the low quantity and high cost of autologous ADSCs extraction, we plan to isolate and purify exosomes from ADSCs and explore the role of exosomes in the treatment of POI. Furthermore, we may be able to determine the paracrine effect of ADSCs in restoring ovarian function. Third, we have just completed a study using POI mouse models. Considering the vast differences between humans and animals, we are planning to treat POI with autologous MLT-ADSCs in a large animal model more similar to the pathophysiology of human ovary.

## Conclusion

This study demonstrated that both autologous ADSCs and autologous 5 µM MLT-ADSCs could repair chemotherapy-induced POI. Since MLT can promote the proliferation and secretion of ADSCs, autologous 5 µM MLT-ADSCs have significant advantages in restoring ovarian function. Moreover, the SIRT6/NF-κB signal pathway might be directly linked with the mechanism of ADSCs in the treatment of POI. The findings may provide a basis for further research and application. Overall, our study provides pre-limited information on the protective effect of 5 µM MLT-ADSCs in the treatment of POI, which might provide a new potential cell transplantation treatment plan for POI patients.

## Data Availability

The data that support the findings of this study are available from the corresponding author upon reasonable request.

## Abbreviations

POI: Premature ovarian insufficiency
ADSCs: Adipose-derived stem cells
MLT: Melatonin
MLT-ADSCs: MLT-preconditioned autologous ADSCs
SIRT6: Sirtuin 6
NF-κB: Transcription factor nuclear factor kappa B
ELISA: Enzyme-linked immunosorbent assay
IHC: Immunohistochemical
qRT-PCR: Quantitative real-time polymerase chain reaction
HRT: Hormone replacement therapy
MSCs: Mesenchymal stem cells
CTX: Cyclophosphamide
BUS: Busulfan
E2: Estradiol
FSH: Follicle-stimulating hormone
H&E: Hematoxylin and eosin
BDNF: Brain-derived neurotrophic factor
VEGF: Vascular endothelial growth factor
HGF: Hepatocyte growth factor
PBS: Phosphate-buffered saline
FBS: Fetal bovine serum
PrFs: Primordial follicles
PF: Primary follicles
SF: Secondary follicles
AF: Antral follicles
IL-1β: Interleukin-1β
